# A Brief Review on The Molecular Basis of Medullary Thyroid Carcinoma

**DOI:** 10.22074/cellj.2016.4715

**Published:** 2016-09-26

**Authors:** Masoumeh Mohammadi, Mehdi Hedayati

**Affiliations:** Cellular and Molecular Endocrine Research Center, Research Institute for Endocrine Sciences, Shahid Beheshti University of Medical Sciences, Tehran, Iran

**Keywords:** Medullary Thyroid Carcinoma, RET Proto-Oncogene, miRNA

## Abstract

Approximately 5-10% of all thyroid cancers are medullary thyroid carcinomas (MTC). MTC is mainly sporadic in nature, but 20-30% of cases are hereditary. Genetic testing for hereditary MTC is very important for the patient and his family, but the patients must be receiving appropriate genetic counseling. About 98% of patients with hereditary MTC have germline mutations in exons 10, 11, 13, 14, 15, 16 and intron 16 of the REarrangement during transfection (RET) proto-oncogene, but the etiology of the more frequent sporadic form of MTC (sMTC) is not well understood. Recently, it has been reported that apparently sporadic MTC may involve point mutations in BRAF and RAS genes, with an overall prevalence of almost 10%. Also alteration and abnormal expression of miRNA has been described in MTC. In this review, we attempted to mention some mutations and molecular changes in sporadic and hereditary MTC pathogenesis.

## Introduction

Medullar thyroid carcinoma arises from the Calcitonin (CT)-producing parafollicular C cells of the thyroid and was first described by Hazard in 1959. Medullary thyroid carcinomas (MTC) accounts for 5 to 10% of all thyroid carcinomas (1,2). It is important to recognize, because its management is different from follicular thyroid tumors (3). MTC is mainly sporadic and only approximately 20-30% of cases are the hereditary form of the disease. The hereditary forms of MTC were first reported in 1961, in a pair of young siblings whose mother had died previously following surgery for thyroid cancer (3,4). 

The hereditary forms of MTC, referred to as 'multiple endocrine neoplasia type 2' (MEN 2), characterized by MTC in combination with pheochromocytoma and hyperparathyroidism (MEN 2A), or MTC in combination with pheochromocytoma, multiple mucosal neuromas and Marfanoid habitus (MEN 2B). The occurrence of familial MTC (FMTC) in the absence of other neoplasia is also possible (5,6). All these disorders are transmitted as an autosomal-dominant trait (7,9). Hereditary MTC occurs, in 60% of cases, with either, multiple endocrine neoplasia type 2A and 5% of MEN type 2B, or 35% of familial medullary thyroid carcinoma (9,11). 

About 20 years ago it was recognized that the genetic cause was mutations of the REarranged during transfection (RET) proto-oncogene (12,13). Since that time, the utility of genetic testing for RET mutations has been to confirm the diagnosis of MEN 2. In fact detection of RET mutations in MEN 2 provides a paradigm for genetically guided patient management, genotype-phenotype correlations, patient and family screening and long-term follow-up (9,14). Also early detection of RET mutation can provide an opportunity for therapeutic intervention prior to advanced disease. In addition to the 20-30% of hereditary MTC cases that have germline mutation in the RET proto-oncogene, about 4-10% of patients with sporadic MTC also have germline mutations in the RET protooncogene, that can consequently be inherited in the future. Therefore, all MTC patients should undergo genetic testing for RET proto-oncogene mutations (15,16). Genetic testing for hereditary MTC is very important for the patient and his family, but the patients must be receiving appropriate genetic counseling. If patients are found to be positive for a RET mutation, other family members need to be identified and should undergo genetic testing. 

The etiology of the most frequent sporadic form of MTC is not well understood, although somatic RET mutations have been detected in up to 43% of patients (17). Recently, it has been reported that apparently sporadic MTC may involve point mutations in BRAF and RAS genes (with an overall prevalence of almost 10%) (15,18,19). It is noteworthy that all these new MTC genes are involved in RET-activated pathways (20). 

Other genetic changes that have been described in MTC are the abnormal expression of non-coding micro RNA (21). A miRNA is a small non-coding RNA molecule containing about 22 nucleotides, whose functions are RNA silencing and post-transcriptional regulation of gene expression (22). MiRNAs are expressed in a tissue-specific manner and with different profiles. Their expressions are differing between normal and neoplastic tissues and between tumors with distinct biological properties (21,23,24). Also, often in human cancer, expression of genes encoding factors involved in miRNA biogenesis (DICER, DROSHA, DCGR8, and XPO5) deregulate and correlate with aggressive clinical behavior (25). 

## REarranged during transfection proto-oncogene

In 1985, Takahashi et al. (26) identified the RET oncogene. They transfected NIH 3T3 mouse fibroblast cells with total DNA from a human lymphoma and obtained one clone which in secondary transformants yielded more than a 100-fold improvement in transformation efficiency, suggesting that there was a new transforming gene. Subsequent analysis revealed that the new transforming gene was derived by DNA rearrangement during transfection from normal human sequences of the RET locus. 

**Fig.1:**
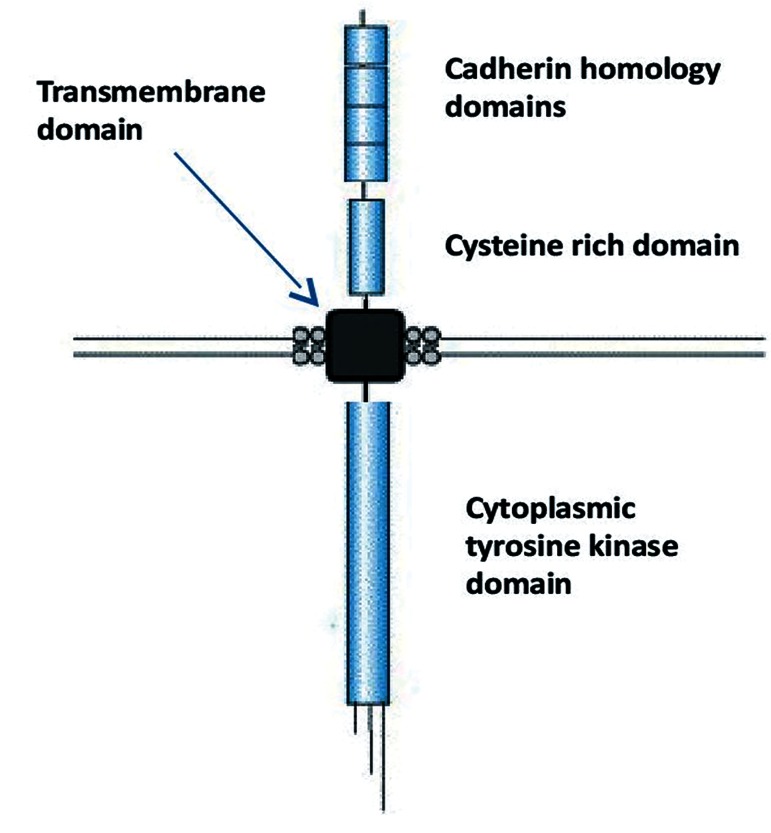
REarrangement during transfection (RET) structure. RET protein composed a cytoplasmic tyrosine kinase domain, a transmembrane domain, and cysteine rich domain.

Thus, this gene was named "REarranged during Transfection" or RET (11,26). Identification of the RET proto-oncogene has had a profound impact on cancer research and developmental biology research (27,28). The proto-oncogene RET is composed of 21 exons that are located on chromosome 10 (10q11.2) and encode the RET protein (170 KDa). This protein is composed of three functional domains, including a cytoplasmic tyrosine kinase domain, a transmembrane domain, and cysteine rich domain (Fig.1). 

**Fig.2:**
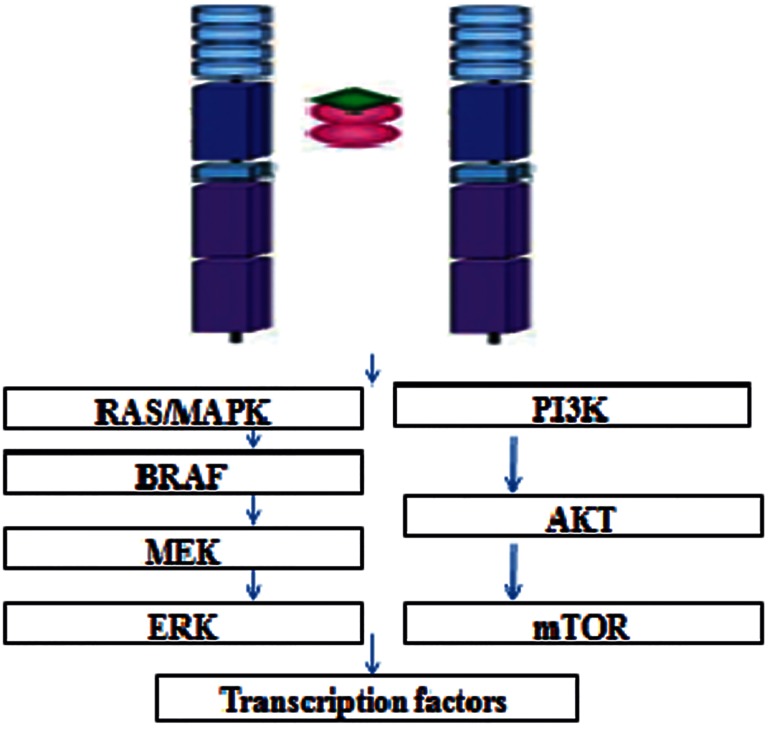
RET signaling. RET signaling leads to the activation of the RAS/MAPK and the PI3K/Akt pathways. RET; REarranged during transfection, MAPK; Mitogen-activated protein kinase, BRAF; Proto-oncogene B-Raf, MEK; Is a member of the MAPK signaling cascade, ERK; Extracellular signal-regulated kinases, PI3K; Phosphatidylinositol 3′ kinase, AKT; Is an oncogene that encode serine/threonine-protein kinase, and mTOR; Mechanistic target of rapamycin.

The extracellular domain contains four cadherin like repeats and a cysteine rich region that binds to ligands. Ligands are glial cell line-derived neurotrophic factor (GDNF) and related molecules, including neurturin (NRTN), artemin (ARTN) and persephin (PSPN) (29,30). The intracellular domain contains two tyrosine kinase subdomains that are involved in several intracellular signal transduction pathways (31). RET activation is mediated by these neurotrophic factors through a unique receptor system, consisting of glycosyl-phosphatidyl-inositol-anchored co-receptor (GFRα1-4) (32,33). RET signaling leads to the activation of the RAS/ mitogen-activated protein kinase (MAPK) and the phosphatidylinositol 3´ kinase (PI3K)/Akt pathways which have key roles in survival, proliferation, differentiation, and migration of the enteric nervous system progenitor cells, as well as the survival and regeneration of kidney cells (Fig.2) (28,34,35). 

## Genetic abnormalities in medullary thyroid carcinomas

### REarranged during Transfection somatic mutations 

Most somatic mutations of the RET proto-oncogene have been identified in codon 918 and occur in 25-33% of sporadic MTC cases (6,17). Other rare mutations in codons 618, 634, 768, 804 and 883, and partial deletion of the RET gene have been identified in a few tumors (6). 

### REarranged during transfection germline mutations

Hereditary MTC is caused by germline autosomal dominant gain of function mutations in the RET proto-oncogene. 

About 98% of patients with hereditary MTC (MEN 2A, MEN 2B and FMTC) have germline mutations. The germline mutations in these patients are in exons 10, 11, 13, 14, 15 and 16 of the RET gene. In most cases mutations causing MEN 2A affect the cysteine rich extracellular domain, each converting a cysteine to another amino acid in codons 609, 611, 618 and 620 of exon 10. The most common mutation, accounting for 80% of these patients, affects codon 634 of exon 11 (2,36,37). 

In about 95% of patients with MEN 2B, a single mutation that converts methionine to threonine at codon 918 of exon 16 has been identified (36). Other rare mutations associated with MEN 2B involve codons 883 and 904 of exon 15, codons 691; also 634 of exon 11; and codon 838 of exon 14 in the intracellular domain of the gene (2,38). 

In FMTC, mutation at codon 620 of exon 10 has been detected in 95% of all cases in addition to rare mutations in codons 611 and 618 of exon 10 (39). Other rare mutations associated with FMTC involve codons 630, 631, 634, 649, 666 and 686 of exon 11; codons 768, 776, 790, 791 of exon 13; codons 804, 844, 833 and 819 of exon 14; codons 866 and 891 of exon 15; and codon 918 of exon 16. In the intracellular domain of the gene mutation in intron of 16 has also been detected (39,41). Most of these mutations involve the extracellular cysteine residue (Table 1) (16). 

Sporadic and hereditary forms of MTC have similar clinical presentations, therefore people with isolated MTC should be offered germline testing for hereditary forms of MTC (41). 

The American Thyroid Association (ATA) has classified MTC risk based on genotype phenotype (14). The different mutations are classified into four risk categories according to the aggressiveness of the disease. Level A mutations carry the least risk, level B and C mutations carry a lower risk of aggressive MTC and level D mutations carry the highest risk for MTC and are associated with highest risk of metastases and mortality and onset in youngest age. Children with MEN 2B phenotype are at ATA level D risk and in the case of early development of MTC should have a thyroidectomy within the first year of life. Children with codon 634 mutations are at ATA level C risk and so also at higher risk and thyroidectomy should be carried out before 5 years of age. Patients with mutations at codons 768, 790, 791, 804, 891 are at ATA level A and patients with codons 609, 611, 618, 620, 630 are in ATA level B so risk of MTC is moderate. In these cases, if there is a less aggressive MTC family history with normal basal stimulated serum calcitonin and normal neck ultrasound; prophylactic thyroidectomy be delayed beyond the age of 5 years. This system may be used to individualize the treatment. 

## REarrangement during transfection (RAS) and proto-oncogene B-Raf (BRAF) mutations

Unlike RET mutations in hereditary MTC, in sporadic MTC, the genetic mutations and molecular alterations are not well established. Thus the pathogenesis of sporadic RET negative MTC is poorly understood. Recently, it has been reported that apparently sporadic MTC may involve point mutations in BRAF and RAS genes (with an overall prevalence of almost 10%) (15,18,19). 

The RAS gene family encodes three homologous isoforms NRAS, HRAS, and KRAS, respectively located on chromosomes 1, 11 and 12. All of them contain 4 exons and encode 21-kDa membrane-associated proteins that play a role in the transduction of signals from receptors (tyrosine kinase and G protein coupled) to adenylate cyclase (MAPK and PI3K/AKT) signaling pathways (40,42). Their activity is regulated by GTP-mediated hydrolysis of activated GTP bound RAS to inactivated GDP bound RAS (43). Point mutations of RAS either increase affinity for GTP or inhibit autocatalytic GTPase function, both mechanisms leading to incorrect activation of MAPK and PI3/ AKT signaling pathways (44,45). 

In thyroid cancer Moura et al. reported that 56% of RET negative sporadic MTC cases had HRAS mutations and 12% had KRAS mutations. However, only one HRAS mutation was found in one case among 40 patients with RET positive sporadic MTC (18,46). Furthermore, a genotype study performed by Goutas et al. (19) in sporadic MTC carcinoma cases reported that KRAS codon 12 mutations were found in 40.9% of the MTC cases, the BRAF V600E mutation in 68.2%. However, no significant association between KRAS and BRAF mutations and clinic-pathological parameters was found. BRAF mutations are more frequently seen in older patients with papillary thyroid cancer (47) and occur rarely in follicular or medullary thyroid carcinomas (48). Consequently there are few studies about BRAF mutation in MTC. 

These findings support the potential role of RAS and BRAF in the pathogenesis of medullary thyroid cancer and suggest that evaluation of RAS mutations in sporadic RET negative MTC patients can be useful to select targeted therapies (18). 

**Table 1:** RET germline mutations in hereditary MTC forms


Exon	FMTC	MEN2A	MEN2B

10	Codon620	Codons609,611,618,620	
11	Codons630,631,634,649,666	Codon634	Codons691,634
13	Codons768,776,790,791		
14	Codons819,833,804,844		Codon838
15	Codons866,891		Codons883,904
16	Codon918		Codon918

RET; REarrangement during transfection, MTC; Medullary thyroid carcinomas, FMTC; Familial MTC, and MEN; Multiple endocrine neoplasia.

### MicroRNA profile in medullary thyroid cancer

MicroRNAs (miRNA) are involved in the pathogenesis of several human cancers, especially MTC. During the last few years, studies on miRNA and cancer have been initiated. MiRNA dysregulation is common in cancer cells and the pattern of miRNA expression can be correlated with cancer type, stage, and other clinical variables, so miRNA profiling can be useful for cancer diagnosis and prognosis. Currently, in addition to disease diagnostics, miRNAs use for therapeutic especially in cancer therapy that is out of this discussion. 

**Fig.3:**
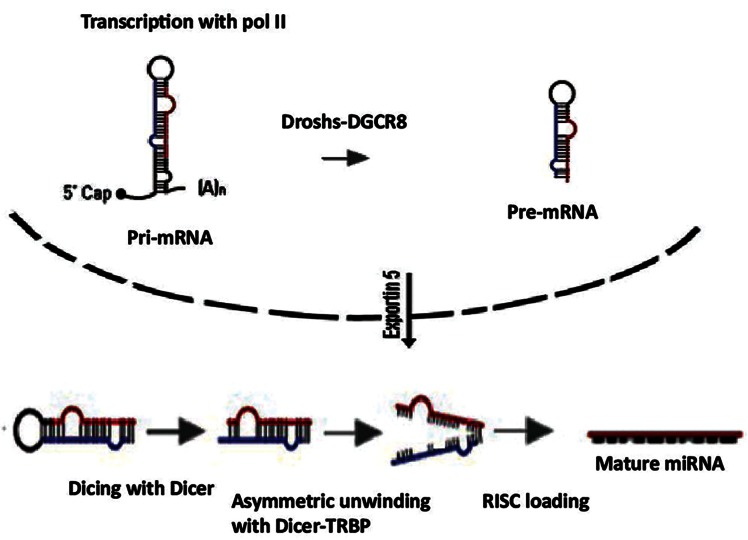
MicroRNA genes are transcribed by RNA polymerase II as pri-microRNA that is processed by a protein complex containing the RNase III enzyme, to form pre-microRNA. Pre-microRNA is processed by a second RNase III enzyme (DICER) to form a mature microRNA with approximately 20-22 nucleotides. The mature microRNAs with ribonuclear particles are incorporated to form the RNA induced silencing complex (RISC) which mediates gene silencing.

MiRNAs are small non-coding RNAs that play key roles in the regulation of gene expression through two principal mechanisms: degradation of mRNAs or inhibition of mRNA translation efficiency (Fig.3). 

Aberrant expression of miRNAs can occur through a number of different mechanisms such as, genomic abnormality, epigenetic factors, transcriptional regulation and regulation at miRNA processing steps (49). 

There is a limited numbers of studies that have evaluated the role of miRNAs in MTC. According to these studies, miRNAs play a pivotal role in the biology of MTC and could pave the way to new therapies. 

Abraham et al. (50) investigated miRNA profiling to identify prognostic biomarkers and potential therapeutic targets in sporadic and heredity forms of MTC. They reported that 10 miRNAs differentially expressed (4 overexpressed and 6 under expressed) were deregulated in sporadic MTC versus heredity MTC and that among these miRNAs, MiRs-183 and 375 were more overexpressed. They showed knock down of miR-183 in the TT cell line induced decrease cell viability and upregulation of the protein LC3B (as microtubule-binding is associated with autophagy). Therefore they suggested the utilization of these miRNAs could represent as prognostic biomarkers and therapeutic targets. 

Main et al. (51) studied miRNA dysregulation in heredity and sporadic MTC patients to determine the relationship between miRNA profiles and the outcome in MTC, and the relationship between miRNA signatures and RET status in sporadic MTC. They showed a significant overexpression of several miRNAs (miR-127, miR154, miR-224, miR-323, miR-370, miR-183, miR-375, and miR-9) in MTC sample. Also miR-127 levels were observed to be lower in sporadic MTC with somatic RET mutations in comparison to sporadic MTC without RET mutations. They showed that in both sporadic MTC and heredity MTC, the miR-224 was upregulated in lower stages at diagnosis and could represent a prognostic biomarker associated with a better outcome in MTC patients. 

In another study Nikiforova et al. (21) investigated expression patterns of miRNA in all major types of thyroid tumors, to explore the utility of miRNA profiling, for the preoperative diagnosis of thyroid nodules, in fine-needle aspiration biopsy (FNAB) samples and surgically removed thyroid neoplastic and non-neoplastic samples. They showed that profiles of miRNA expression were not markedly different between C cell derived thyroid carcinoma and all other thyroid tumors that derive from follicular cells. However, various histopathological types of thyroid tumors (derived from the same cell) have different miRNA profiles, which reflect the specific oncogenic mutations found in these tumors. 

In addition to miRNA deregulation, expression of gene encoding factors involved in miRNA biogenesis is deregulated in human cancer. According to previous studies, master miRNA regulators DROSHA, DICER and DGCR8 were reduced in several tumors. 

In this regard, there are few studies about miRNA expression and expression of genes involved in miRNA biogenesis, in MTC. 

Puppin et al. (25) studied, expression of four genes [DICER, DROSHA, DCGR8, and Exportin 5 (XPO5)] involved in miRNA biogenesis in MTC specimens. They showed DICER, DGCR8, and XPO5 mRNA levels were significantly overexpressed in MTC with RET mutations. They suggested genes involved in miRNA biogenesis could represent markers for targeted therapies in the treatment of mutated RET. 

As the biological role of miRNA dysregulation in thyroid carcinogenesis remains poorly understood, it is necessary to continue the evaluation of miRNA in thyroid cancers, especially in MTC. 

## Conclusion

Advances have been made in understanding the molecular pathogenesis of MTC. The RET proto-oncogene has been introduced as the susceptibility gene for hereditary MTC but in sporadic MTC, the genetic mutations and molecular alterations are not well established. Although recently, point mutations in BRAF and RAS genes and also abnormal expression of non-coding miRNA have been described for sporadic MTC for therapeutic goals, more work is needed on the molecular pathogenesis of MTC and on the genetic defects and altered cellular signaling pathways involved in the tumorigenesis of human MTC. 
